# Long Non-coding RNAs, Novel Culprits, or Bodyguards in Neurodegenerative Diseases

**DOI:** 10.1016/j.omtn.2017.12.011

**Published:** 2017-12-22

**Authors:** Ding-Qi Wang, Peng Fu, Chengye Yao, Ling-Shuang Zhu, Tong-Yao Hou, Jian-Guo Chen, Youming Lu, Dan Liu, Ling-Qiang Zhu

**Affiliations:** 1 Department of Pathophysiology, Key Lab of Neurological Disorder of Education Ministry, School of Basic Medicine, Tongji Medical College, Huazhong University of Science and Technology, Wuhan 430030, PRC; 2 Department of Neurosurgery, Union Hospital, Huazhong University of Science and Technology, Jiefang Avenue No. 1277, 430022 Wuhan, PRC; 3 Department of Neurology, Union Hospital, Tongji Medical College, Huazhong University of Science and Technology, Wuhan 430022, PRC; 4 Department of Pharmacology, School of Basic Medicine, Tongji Medical College, Huazhong University of Science and Technology, Wuhan 430030, PRC; 5 The Institute for Brain Research, Collaborative Innovation Center for Brain Science, Huazhong University of Science and Technology, Wuhan 430030, PRC; 6 Department of Medical Genetics, School of Basic Medicine, Tongji Medical College, Huazhong University of Science and Technology, Wuhan 430030, PRC

**Keywords:** long non-coding RNAs, lncRNA, neurodegenerative diseases, Alzheimer’s disease, Huntington’s disease, amyotrophic lateral sclerosis

## Abstract

Long non-coding RNA (lncRNA) is a kind of non-coding RNA (ncRNA), with a length of 200 nt to 100 kb, that lacks a significant open reading frame (ORF) encoding a protein. lncRNAs are widely implicated in various physiological and pathological processes, such as epigenetic regulation, cell cycle regulation, cell differentiation regulation, cancer, and neurodegenerative diseases, through their interactions with chromatin, protein, and other RNAs. Numerous studies have suggested that lncRNAs are closely linked with the occurrence and development of a variety of diseases, especially neurodegenerative diseases, of which the etiologies are complicated and the underlying mechanisms remain elusive. Determining the roles of lncRNA in the pathogenesis of neurodegenerative diseases will not only deepen understanding of the physiological and pathological processes that occur in those diseases but also provide new ideas and solutions for their diagnosis and prevention. This review aims to highlight the progress of lncRNA research in the pathological and behavioral changes of neurodegenerative diseases. Specifically, we focus on how lncRNA dysfunctions are involved in the pathogenesis of Alzheimer’s disease, Parkinson’s disease, Huntington’s disease, and amyotrophic lateral sclerosis.

## Main Text

In the human genome, less than 2% of the transcripts encode proteins, and the remaining 98%–99% are non-coding RNAs (ncRNAs) .^1^ ncRNAs can be divided into two categories: short non-coding RNA (short/small ncRNA) and long non-coding RNA (long ncRNA). No particularly stringent boundary exists between these categories, except for the length of the ncRNA nucleotide sequence. An ncRNA of greater than 200 nt is defined as an lncRNA. In recent years, with the rapid development of RNA sequencing technology and computational methods, lncRNA has been gradually recognized by researchers. lncRNA is widely distributed among animals, plants, yeasts, and even viruses, and we now know that lncRNA can be involved in X chromosome silencing, genomic imprinting, chromatin modification, transcriptional activation, transcriptional interference, epigenetic regulation, and other important regulatory processes.^2^ These molecules are related to cell proliferation and differentiation, metabolism, and other physiological processes, as well as various pathological processes. The mutation and abnormal regulation of lncRNAs plays an important role in many human diseases, such as the occurrence and development of cancer and neurodegenerative diseases.^3^, ^4^, ^5^

Neurodegenerative disease is characterized by the loss of neurons or myelin in the brain and spinal cord, deteriorating over time and leading to dysfunction.^6^, ^7^ The brain and spinal cord comprise neurons that have different functions, such as controlling motion, processing sensory information, and making decisions. The cells of the brain and spinal cord are generally not regenerated; thus, excessive damage can be devastating and irreversible. Neurodegenerative diseases are widespread worldwide. The long course and lack of effective treatment of these diseases impose a heavy burden on individuals, families, and society. In recent years, many studies have shown that lncRNA can participate in the regulation of neurodegenerative disease occurrence and development. Based on existing research results, this paper focuses on lncRNA in several common neurodegenerative diseases. We made a summary of lncRNAs involved in this review (Table 1).

**Table 1:** Dysregulated lncRNAs in Neurodegenerative Diseases

lncRNAs	Description	Associated Disease	Culprit or Bodyguard	Biological Function
BACE1-AS	transcribe from the antisense protein-coding BACE 1 gene	AD	culprit	bind to BACE1 and increase the stability of its mRNA, thereby promoting the synthesis of BACE1 protein, and further increase the production of Aβ of cells
BC200	homologous with rodent BC1 lncRNA; the earliest specific example showed lncRNAs conservation	AD	culprit	induce APP mRNA translation via association with FMRP and then aggregate the accumulation of Aβ in the brain
17A	embedded in the human G-protein-coupled receptor 51 gene	AD	culprit	impair GABAB signaling pathway by decreasing GABAB R2 transcription
NAT-Rad18	transcribed from the antisense of protein coding gene Rad18	AD	culprit	down the expression of DNA repair protein Rad18 and enhance susceptibility to neuronal apoptosis
51A	an antisense transcript of intron 1 of the SORL1 gene	AD	culprit	altering the spliced form of SORL1 mRNA and resulting in Aβ42 accumulation
GDNFOS	transcribed from the opposite strand of GDNF gene	AD	culprit	negatively regulate the expression of GDNF
HttAS_v1	antisense transcript of the Htt gene	HD	bodyguard	reduce endogenous HTT transcript levels
BDNFOS	antisense transcription product of BDNF	HD	bodyguard	upregulates the transcription of BDNF and have a protective effect on neurons
NEAT1	a nuclear-enriched ncRNA essential for the formation and maintenance of paraspeckles	HD	bodyguard	essential for the integrity of the nuclear paraspeckle substructure; increase viability under oxidative stress
HAR1F and HAR1R	antisense transcripts of the first HAR1 gene	HD	bodyguard	involved in neurotransmission, memory structure, and synaptic plasticity in the mature brain
DGCR5	a transcript of DiGeorge critical region 5	HD	bodyguard	include a genome binding site for REST and play an important transcriptional regulatory role in HD
MEG3	the human homolog of the mouse maternally expressed gene Gtl2, the first imprinted gene identified on the mouse distal chromosome 12	HD	culprit	alter gene expression in response to neuronal activity
ABHD11-AS1	homologous with rodent Abhd11os lncRNA	HD	bodyguard	attenuate the toxicity of Htt mRNA
NaPINK1	transcribed from the antisense of PINK1 locus	PD	bodyguard	stabilize PINK1 expression
AS Uchl1	antisense transcript of Uchl1	PD	bodyguard	regulate the expression of UCHL1 at the post-transcriptional level, thus promoting the translation process and increasing protein synthesis
HOTAIR	antisense intergenic RNA transcribed from the HOXC locus	PD	culprit	increase the stability of LRRK2 mRNA and upregulated its expression, thus inducing DA neuronal apoptosis
MALAT1	also known as NEAT2 and is a highly conserved ncRNA highly expressed in neurons	PD	culprit	upregulate a-synuclein expression, thus driving the pathogenesis of PD

### Classification, Origin, and Function of lncRNA

lncRNAs were first found in rat full-length cDNA sequence libraries, usually overlapping or dispersed between transcripts. According to location and context, lncRNA can be divided into five categories, including inter-gene long-chain non-coding RNA (intergenic lncRNA, also known as long intergenic ncRNA or lincRNA, which is produced by the transcription of spacer sequences between genes encoded in the genome), intron long-chain non-coding RNA (intronic lncRNA, which is completely transcribed from introns within a protein-coding gene), sense long-chain non-coding RNA (sense lncRNA, transcribed from the coding strand of a protein-coding gene and partly or completely overlapping the gene exons), antisense long-chain non-coding RNA (antisense lncRNA, transcribed from the non-coding strand of a gene), and bidirectional long-chain non-coding RNA (bidirectional lncRNA, transcribed from the promoter region in two opposite directions) (Table 2).^8^, ^9^

**Table 2:** Classification of lncRNAs

Classification	Description	Example
Intergenic	produced by the transcription of spacer sequences between genes encoded in the genome	XIST, TMEM161B-AS, HAR1, NEAT1, TUG1
Intronic	transcribed from introns within a protein-coding gene	Lnc-OR51B4-3, KCNIP4-IT1
Sense	transcribed from the coding strand of a protein-coding gene and partly or completely overlapping the gene exons	SNHG4
Antisense	transcribed from the non-coding strand of a gene	BACE1-AS, MALAT1, lnc-LRR1, TUSC7
Bidirectional	transcribed from the promoter region in two opposite directions	Hoxa11as, IGF2AS, HOTAIRM1

At present, the source of lncRNA is not very clear. Ponting et al.^10^ thought that lncRNA may have arisen in the following ways: mutations in the open reading frames (ORFs) of protein-coding genes during early evolution, leading to structural disruption and then to lncRNA; chromatin recombination resulting in two originally distant non-transcript fragments coming together to produce lncRNA; duplication of non-coding genes formed by reverse transcription transposition; repetition of a certain sequence, resulting in lncRNA with adjacent repeat sequences; and DNA sequence insertion by a transposable element to produce functional lncRNA. Although many lncRNAs have no common origin, they play similar roles in the regulation of gene expression, and their most important function may be epigenetic regulation of the genome.^10^

lncRNA is located in the nucleus or cytoplasm and lacks the ability to encode proteins. Initially, biologists thought that lncRNA had no biological function and was only the “noise” of genomic transcription, a byproduct of the activity of RNA polymerase II (RNA PII).^11^ However, with further research, people have gradually gained a deeper understanding of lncRNA, its presence in the subcellular structure, and its ability to regulate protein localization and activity. lncRNA can regulate gene expression by recruiting RNA PII or inducing chromatin recombination.^12^ Compared with small ncRNA, lncRNA has a relatively long chain of nucleotides, meaning that the internal folding of lncRNA molecules can form many specific and complex secondary spatial structures; these structures provide multiple sites, allowing interaction with a number of other molecules to perform biological functions involved in regulating growth and development,^13^ cell proliferation and differentiation,^14^ apoptosis,^15^ and other processes. Meanwhile, a variety of clinical diseases are closely related to lncRNA.^16^ Although the mechanisms of lncRNA involvement in various diseases are not fully understood, this study shows that lncRNA is widely involved in various regulatory mechanisms. lncRNA can be released after being activated by cellular stress and then form complexes with the ribosome to regulate gene expression. Molecular studies have suggested that lncRNA may work in the following modes. (1) Interacting with chromatin: lncRNA regulates the upstream promoter regions of coding genes by mediating chromatin remodeling and histone modification and then affects the expression of related genes. (2) Interacting with proteins: lncRNA can be used as a guiding molecule for a single protein or it can act as a scaffold molecule for two or more proteins to synthesize a protein complex and then recruit the complex to corresponding locations in coding genes to regulate the expression of downstream genes. In addition, lncRNA can also be used as a bait molecule for proteins. It can transfer proteins that are bound to the genome and change their intracellular localization, thereby inhibiting gene transcription and expression. (3) Interaction with other RNAs: many specific microRNA (miRNA) recognition sites are present in lncRNA. On the one hand, miRNAs can affect the stability of lncRNA *in vivo*, mediating the degradation of lncRNA and thus regulating its cell biological functions. On the other hand, lncRNA can act as a bait molecule for miRNA, targeting miRNA by target mimicry and inhibiting its further action, thus indirectly regulating the expression of miRNA target genes. In addition, lncRNA can act as an endogenous competitor of miRNA by binding miRNA-binding sites on mRNA.^17^, ^18^ Moreover, lncRNA can regulate mRNA at the post-transcriptional level by mRNA transcription inhibition, splicing, and degradation.^19^

### lncRNA and Alzheimer’s Disease

Alzheimer’s disease (AD) is the most common cause of dementia. Deposition of beta-amyloid proteins (amyloid beta-peptide [Aβ]) in brain tissue, which can induce senile plaques, is one of the important factors underlying AD pathogenesis.^20^

Aβ is a normal metabolite generated by the hydrolysis of β-amyloid precursor protein (APP). β-site APP cleaving enzyme 1 (BACE1), a membrane-bound aspartic protease, is involved in the processing of APP and cleaves it to Aβ. BACE1-AS (BACE1-antisense) is an antisense transcript of BACE1. The expression of BACE1-AS in the brain of AD patients is upregulated, suggesting that BACE1-AS may play a role in the pathogenesis of AD.^21^, ^22^ Studies have shown that BACE1-AS can bind to BACE1 mRNA, increase the stability of the latter, thereby promoting the synthesis of BACE1 protein,^21^ and further increase the production of Aβ of cells. Faghihi et al.^23^ found that the microRNA miR-485-5p, sharing the same binding sites with BACE1 to BACE1 mRNA, inhibited the expression of BACE1 through competition.

Brain cytoplasmic (BC) RNA is another lncRNA that causes AD and brain aging. Mouse BC1 RNA and human BC200 RNA are lncRNA transcripts that are transported as ribonucleoprotein particles to the dendritic processes and bind to poly(A)-binding protein (PABP1), a regulator of translation initiation, thus regulating gene expression at the translational level.^24^ BC200 can interact with some RNA-binding proteins involved in aberrant transport of mRNA, leading to abnormal protein localization. Overexpression of BC200 in AD and the aging brain may cause synaptic/dendritic degeneration. Mus et al.^25^ found that the level of BC200 in the AD-affected brain region Brodmann area 9 was higher than that in the normal brain, and the relative level of BC200 RNA in the affected region increased in parallel with AD severity. Simultaneously, they found that in the advanced stage of AD, BC200 RNA often assumed a clustered perikaryal localization, indicating that dendritic loss is accompanied by somatic overexpression. Mus et al. concluded that this was a stress compensation mechanism initiated by dendritic sprouting and remodeling in AD degenerative lesions; they also thought that the abnormal localization and distribution of BC200 might be responsible for the cause and course of the disease. Whether BC200 overexpression in brain tissue is the cause of AD or an accompanying compensatory response will require further testing and confirmation.^26^ Recently, Zhang et al.^27^ showed that BC1 induces APP mRNA translation via association with a fragile X syndrome protein (FMRP). Inhibition of BC1 or BC1-FMRP association in AD mice blocks the aggregation of Aβ in the brain and improves spatial learning and memory. Expression of exogenous BC1 in mouse excitatory pyramidal neurons shows the opposite result. This study may provide a novel promising target for AD therapy.

Under inflammatory stimulation, lncRNA-17A can direct G-protein-coupled receptor 51 (GPR51) splicing to produce isoform variant B of the B-type receptor for the neurotransmitter g-aminobutyric acid (GABA; GABAB receptor). Because only the canonical variant, isoform A, contains the complete C-terminal portion needed to interact with GABAB R1 to generate a functional heterodimeric receptor, this alternative splicing causes the synthesis of a non-functional GABAB receptor. In addition, the overexpression of 17A causes an overproduction of Aβ.^28^

**Figure 1:**
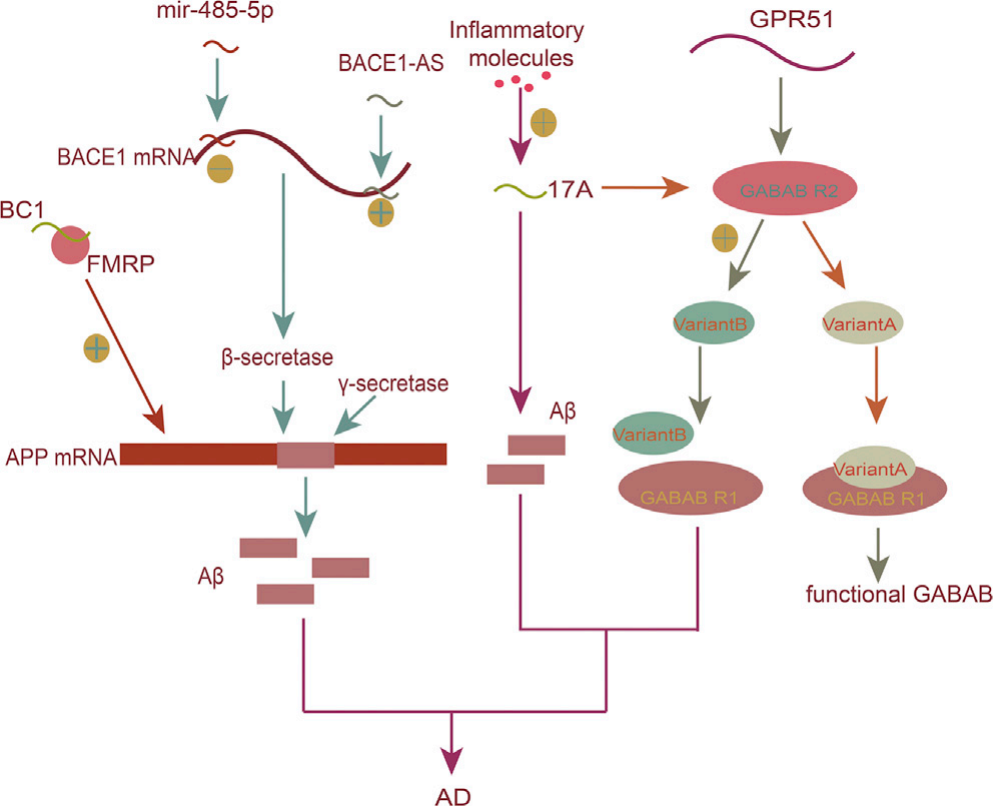
Role of lncRNAs in AD BACE1-AS can bind to BACE1 mRNA, increase the stability of the latter, and promote the synthesis of BACE1 protein, further increasing the production of cell Aβ. miRNA miR-485-5p has an inhibitory effect on BACE1 expression, whereas miRNA miR-485-5p and BACE1-AS have the same binding sites on BACE1 mRNA, so their regulation of BACE1 mRNA expression is antagonistic. BC1 induces APP mRNA translation via association with FMRP. Under inflammatory stimulation, lncRNA-17A can direct GPR51 splicing to produce GABAB receptor isoform variant B, thus causing the synthesis of a non-functional GABAB receptor. In addition, overexpression of 17A causes overproduction of Aβ.

Some lncRNA polymorphisms are associated with AD pathogenesis. For example, rs7990916 (T > C) located in the lincRNA TCONS_00021856/linc-SLITRK5-11 gene showed significant differences between AD patients and a normal control group.^29^ Rad18 is involved in proliferating cell nuclear antigen (PCNA) ubiquitination and plays an important role in DNA repair following nerve injury. After Aβ-induced apoptosis, the Rad18 gene antisense transcription product lncRNA NAT-Rad18, which exerts post-transcriptional control over Rad18, is upregulated, suggesting that it may enhance susceptibility to neuronal apoptosis; excess cell loss may contribute to AD.^30^ lncRNA 51A is an antisense transcript of intron 1 of the sorting protein-related receptor 1 (SORL1) gene, resulting in Aβ42 accumulation by altering the spliced form of SORL1 mRNA.^31^ Zhou et al.^32^ have found 24 upregulated and 84 downregulated lncRNAs in AD patients compared with controls, most being intergenic. Gene set enrichment analysis identified a downregulated lncRNA, n341006, in association with the protein ubiquitination pathway, and a significantly upregulated lncRNA, n336934, linked to cholesterol homeostasis. The lncRNA GDNFOS is a transcriptional product transcribed from the opposite strand of glial-cell-line-derived neurotrophic factor (GDNF) that may negatively regulate the expression of GDNF to promote the course of AD.^33^ Neuroblastoma differentiation marker 29 (NDM29), which is an RNA polymerase (pol) III-transcribed ncRNA, can be promoted by inflammatory stimuli and induces APP synthesis, leading to the increase of Aβ secretion and the process may occur in AD (Figure 1).^34^

Given that aging is also one of the major risks of neurodegenerative diseases, the role of lncRNA in aging cannot be overlooked. Zhang et al.^35^ have identified 97 significantly upregulated and 114 significantly downregulated lncRNA transcripts from the senescence-accelerated mouse prone 8 (SAMP8) and senescence-accelerated mouse resistant 1 (SAMR1) models. These significantly dysregulated lncRNAs were involved in nerve growth factor term, the mitogen-activated protein kinase signaling pathway, and the AD pathway, thus regulating the development of AD.

### lncRNA and Huntington’s Disease

Huntington’s disease (HD), also known as chorea or Huntington’s chorea, is an autosomal dominant neurodegenerative disease characterized by progressive dyskinesias and mental decline.

**Figure 2:**
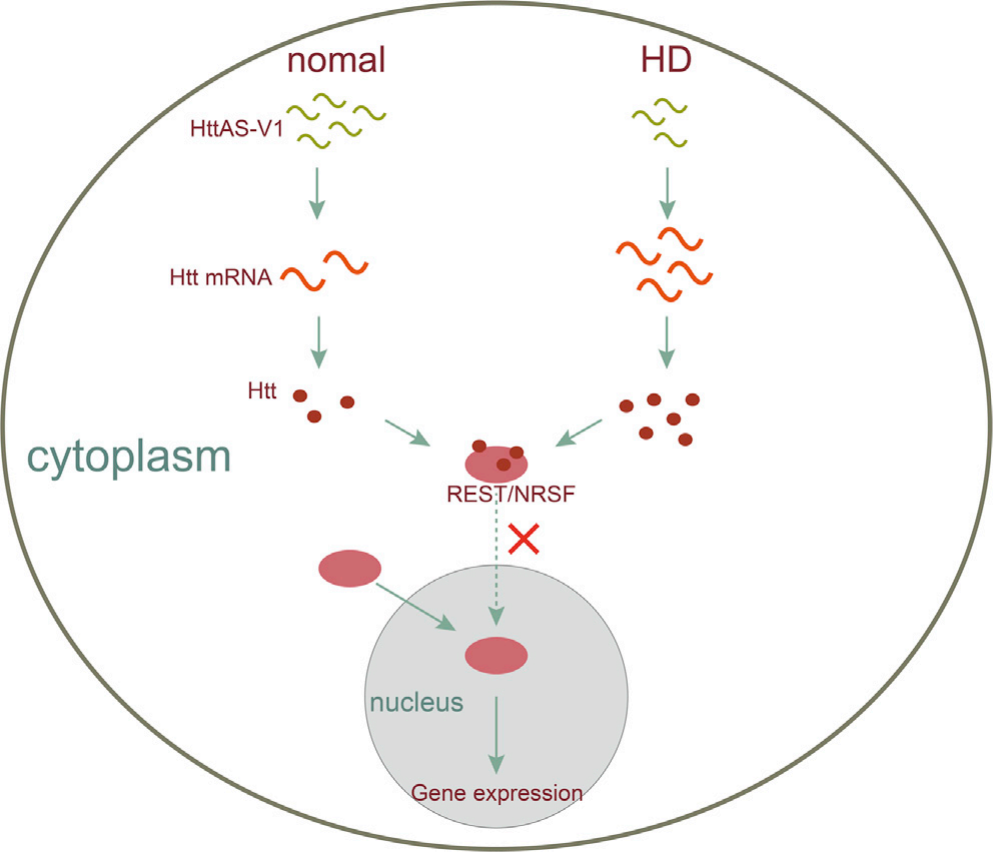
Role of the lncRNA HttAS-v1 in HD The expression of HttAS-v1 is decreased in HD patients, resulting in high expression of Htt mRNA. Htt is involved in the translocation of REST/NRSF into the nucleus, retaining REST/NRSF in the cytosol and thereby preventing REST/NRSF target gene repression.

In HD, the Huntingtin (Htt) protein accumulates gradually in cells, forming large molecules that accumulate in the brain, affecting the function of nerve cells, and resulting in the progressive death of the striatum and cortical nerve cells. lncRNA HttAS_v1 is an antisense transcript of the Htt gene and is expressed at a low level in the frontal cortex of HD patients, leading to high expression of Htt mRNA, which in turn promotes HD pathogenesis.^36^ Htt modulates the nuclear translocation of the transcriptional repressor RE1 silencing transcription factor/neuron-restrictive silencer factor (REST/NRSF). Mutant Htt promotes abnormal nuclear-cytoplasmic transport of REST/NRSF and then leads to abnormal expression of REST target genes, including protein-coding and non-coding genes (Figure 2).^37^, ^38^

BDNFOS is an antisense transcription product of brain-derived neurotrophic factor (BDNF), which upregulates the transcription of BDNF and the translation of BDNF-mRNA in HD. Small interfering RNA (siRNA) treatment with BDNFOS can induce Htt expression. BDNFOS has a protective effect on neurons and improves the HD phenotype,^39^ which is of some positive significance in the prevention and treatment of HD.

NEAT1 (nuclear paraspeckle assembly transcript 1) is a nuclear-enriched ncRNA essential for the formation and maintenance of paraspeckles, which are subnuclear bodies found in mammalian cells.^40^ Sunwoo et al.^4^ found that NEAT1 levels increased in R6/2 mice and HD patients. To determine the biological effects of NEAT1 on neuronal survival, they transfected neuro2A cells with NEAT1 short isoform vector and subjected them to H2O2-induced damage. The NEAT1-transfected cells showed increased viability under oxidative stress, confirming that NEAT1 upregulation contributes to neuroprotective mechanisms against neuronal damage, rather than to the pathology of neurodegenerative diseases.

To detect ncRNAs involved in the pathogenesis of HD, one study examined the ncRNA expression profile of human HD brain tissue and found that the expression of the lncRNAs HAR1F and HAR1R, which are antisense transcripts of the first human accelerated region 1 (HAR1) gene, was significantly reduced in the striatum. These lncRNAs are involved in neurotransmission, memory structure, and synaptic plasticity in the mature brain, and their aberrant expression is also associated with HD.^41^ The same study also demonstrated that abnormal REST nuclear-cytoplasmic exchange in the striatum of HD led to effective transcriptional repression of HAR1.^42^ The lncRNA DGCR5 is a transcript of DiGeorge critical region 5 (DGCR5) and includes a genome-binding site for REST, which plays an important transcriptional regulatory role in HD.^43^ The expression of the lncRNA MEG3, encoded by maternally expressed gene 3 (MEG3), was downregulated in HD brain tissue, and its downregulation was also inhibited by REST.^44^ There is much disparate research showing that MEG3 may be an important epigenetic regulator in the developing and adult human brain, which may stably alter gene expression in response to neuronal activity. If so, the downregulation of MEG3 in HD could plausibly have serious consequences. Overexpression of the lncRNA Abhd11os (ABHD11-AS1 in humans) attenuates the toxicity of Htt mRNA in murine models of HD. Studies have shown that knocking out the Abhd11os gene in the HD mouse model produces neurotoxicity, whereas overexpression of Abhd11os has a neuroprotective effect.^45^

### lncRNA and Parkinson’s Disease

Parkinson’s disease (PD) is a chronic progressive movement disorder caused by loss of brain dopaminergic neurons. PD is a common neurodegenerative disease in the elderly, with clinical symptoms such as resting tremor, muscle tension instability, and posture instability.

Loss or overexpression of PTEN-induced kinase 1 (PINK1) results in abnormal dopamine release and impaired motor function.^46^ NaPINK1 is a human-specific ncRNA transcribed from the antisense orientation of the PINK1 locus that can stabilize PINK1 expression. NaPINK1 silencing results in decreased PINK1 expression in neurons.^47^

The ubiquitin carboxy-terminal hydrolase L1 gene (Uchl1) has been shown to be closely related to brain function and neurodegenerative diseases. An antisense transcript of Uchl1, AS Uchl1, can regulate the expression of UCHL1 at the post-transcriptional level and enhance contact between post-transcriptional mRNA and polysaccharides, thus promoting the translation process and increasing protein synthesis. This activity has been disrupted in patients with familial PD, and studies have reported losses of UCHL1 activity in many other neurodegenerative diseases.^48^, ^49^ Regulation of Uchl1 expression can provide new ideas for PD treatment.

Previous studies have showed that a-synuclein aggregation is the driving force in PD pathogenesis.^50^ Metastasis associated lung adenocarcinoma transcript 1 (MALAT1), also known as nuclear-enriched abundant transcript 2 (NEAT2), is a highly conserved ncRNA highly expressed in neurons.^39^, ^51^ Recently, research has shown MALAT1 overexpression upregulated a-synuclein expression, whereas inhibition of MALAT1 downregulated its expression only in the protein level rather than the mRNA level. Giving β-asarone, the major ingredient of Acorus tatarinowii Schott, can decrease the expression levels of MALAT1 and a-synuclein in the midbrain tissue of PD mice, suggesting β-asarone as a potential therapeutic agent for PD.^52^

**Figure 3:**
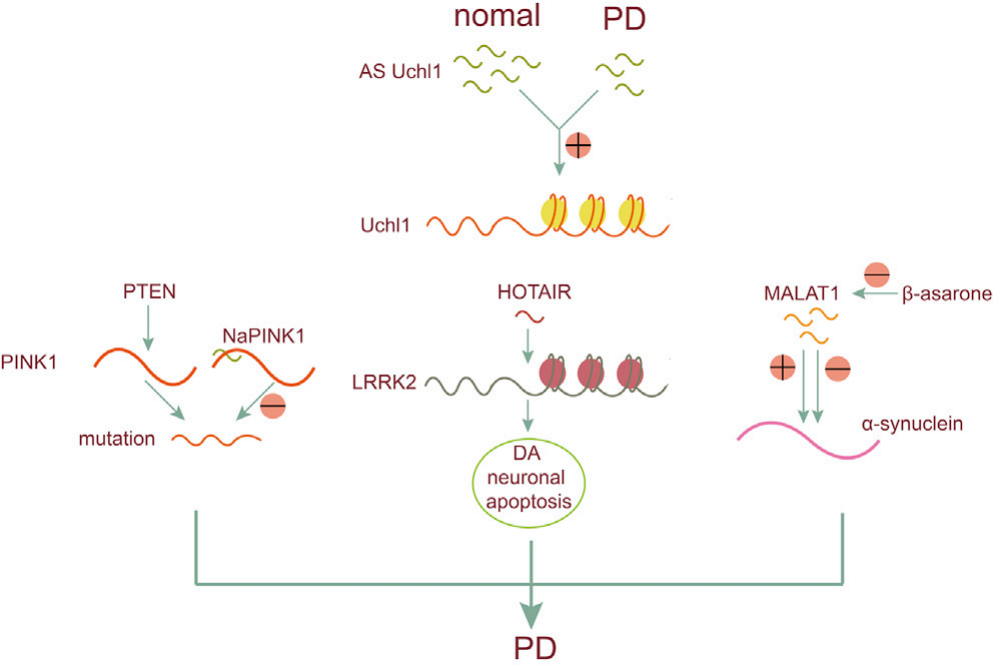
Role of lncRNAs in PD NaPINK1 can stabilize PINK1 expression. AS Uchl1 can regulate the expression of UCHL1 at the post-transcriptional level and enhance contact between post-transcriptional mRNA and polysaccharides, thus promoting the translation process and increasing protein synthesis. HOTAIR can increase the stability of LRRK2 mRNA and upregulated its expression, thus inducing DA neuronal apoptosis. MALAT1 can upregulate a-synuclein expression, thus driving the pathogenesis of PD.

Leucine-rich repeat kinase 2 (LRRK-2) is thought to be involved in the initiation/development of PD.^53^ Recently, research has shown that HOTAIR (Hox transcript antisense intergenic RNA), an approximately 2.2-kb-long noncoding RNA transcribed from the HOXC locus, is upregulated in a mouse model of PD that is developed by intraperitoneal injection of MPTP. The upregulated HOTAIR can specifically increase the stability of LRRK2 mRNA and upregulated its expression, thus inducing DA neuronal apoptosis (Figure 3).^54^

### lncRNA and Amyotrophic Lateral Sclerosis

Amyotrophic lateral sclerosis (ALS) is a neurodegenerative disease that has no effective therapeutic means at present. ALS is characterized by the progressive paralysis of the limbs and muscles involved in speech, swallowing, and respiration due to the progressive degeneration of spontaneous motor neurons. In 2011, the repeated amplification of a six-nucleotide motif (GGGGCC) in the protein-coding gene C9ORF72 (chromosome 9 ORF 72) became the first identified causative mutation in ALS and front temporal dementia (FTD).^55^, ^56^ Non-coding transcripts have now also been identified at the C9ORF72 locus. The transcription of the C9ORF72 repeats is bidirectional, producing both sense and antisense RNAs,^57^ both of which are elevated in the brains of ALS patients, and these transcripts are localized predominantly in the nucleus.^58^ C9ORF72 antisense lncRNA can inhibit C9ORF72 mRNA, thereby inhibiting gene expression. However, even if the expression of a disease-related gene is corrected in fibroblasts derived from a patient, the disease still cannot be treated.^58^

**Figure 4:**
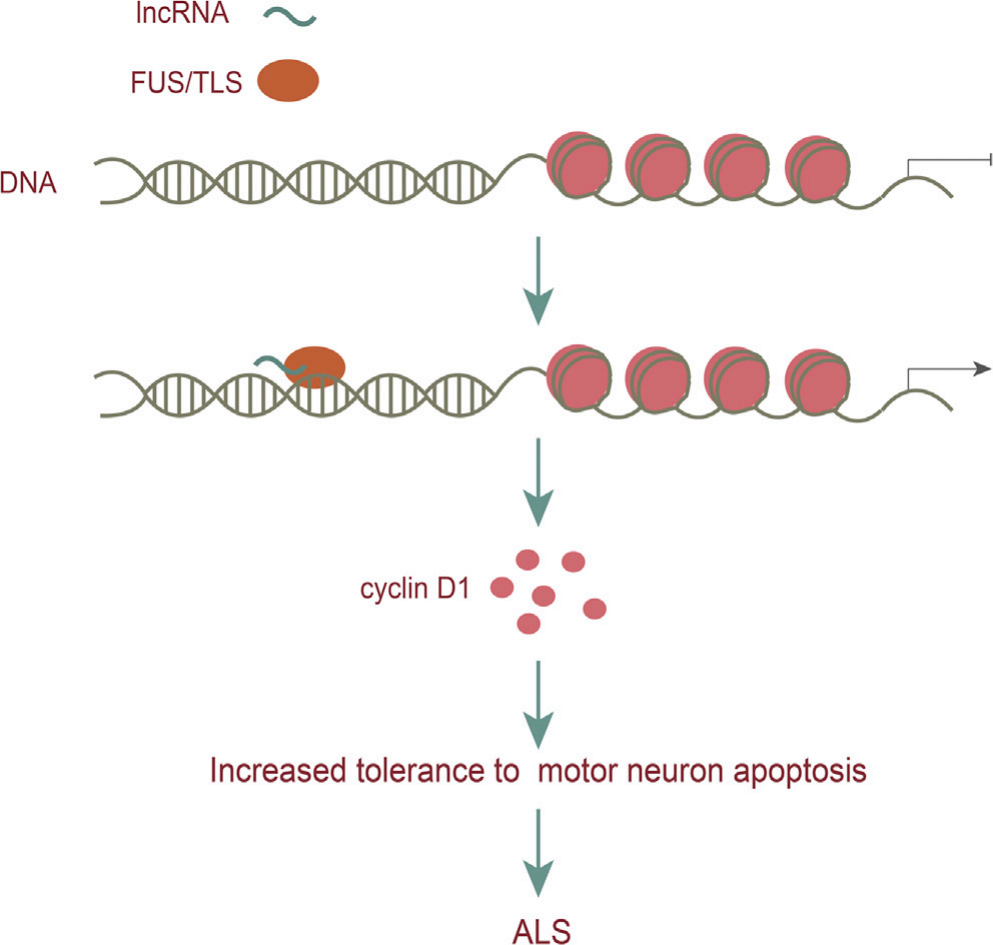
Role of lncRNAs in ALS lncRNA could recruit FUS/TLS to the genomic locus that encodes cyclin D1, where cyclin D1 transcription is repressed in response to DNA damage signals, resulting in increased tolerance to apoptosis signals.

TDP43 (TAR DNA-binding domain protein 43) and FUS/TLS (fused in sarcoma/translated in liposarcoma) are RNA-binding proteins that are predominantly located in the nucleus and play a role in regulating RNA metabolism. Studies have shown that abnormal accumulation of FUS/TLS and TDP43 in the cytosol directly leads to the misfolding of wtSOD1 (wild-type Cu/Zn superoxide dismutase) in non-SOD1 FALS (familial ALS) and SALS (sporadic ALS), which means that they constitute common molecular pathogenesis mechanisms of ALS.^59^ A previous study found that lncRNA could recruit FUS/TLS to the genomic locus that encodes cyclin D1, where cyclin D1 transcription is repressed in response to DNA damage signals, resulting in increased tolerance to apoptosis signals and indicating that lncRNA may play a role in pathological changes in ALS (Figure 4).^60^, ^61^

### Conclusions

With the rapid development of RNA sequencing technology and computational methods, lncRNA has gradually been recognized by researchers, and the current research regarding lncRNA in neurological diseases has made some progress. The expression patterns of most lncRNAs are more tissue specific than those of their respective mRNAs, suggesting that lncRNAs can be used as molecular markers for disease diagnosis and drug design. In view of the specificity effects of certain lncRNAs, targeted therapeutic drugs can be designed to minimize the impact of other unrelated genes to achieve the purpose of personalized treatment. However, the evidence for the association of lncRNAs with neurological disease is mainly due to differences in expression, and only a few functions of lncRNAs have been clearly demonstrated. Dynamic analysis of changes in the body is constrained by available analytical techniques. Along with the specificity of lncRNA itself, such as its numerous species, the small amount of information contained in the original sequences and the limitation of research methods have contributed to the superficiality of our knowledge of the mechanism of lncRNA action and of other regulatory mechanisms. It remains necessary to continue to pursue in-depth research into the effects of lncRNA and other regulatory mechanisms affected by the microenvironment of the body.

## Conflicts of Interest

The authors have declared that there are no conflicts of interest.
